# Editorial: Gerontechnologies for home support

**DOI:** 10.3389/fpsyg.2024.1477507

**Published:** 2024-09-24

**Authors:** Alexander Moreno, Henk Herman Nap, Sumi Helal, Gloria M. Gutman

**Affiliations:** ^1^Department of Psychology, Université de Montréal, Montreal, QC, Canada; ^2^Centre de Recherche de l'Institut Universitaire de Gériatrie de Montréal, CIUSSS du Centre-Sud-de-l'Île-de-Montréal, Montreal, QC, Canada; ^3^Notre-Dame Hospital, Centre Intégré Universitaire de Santé et de Services Sociaux du Centre-Sud-de-l'Île-de-Montréal (CCSMTL), Montreal, QC, Canada; ^4^Vilans - Dutch Expertise Centre on Long-term Care, Utrecht, Netherlands; ^5^Eindhoven University of Technology, Eindhoven, Netherlands; ^6^Department of Computer Science and Engineering, University of Bologna, Bologna, Italy; ^7^Department of Gerontology, Gerontology Research Centre, Simon Fraser University, Vancouver, BC, Canada

**Keywords:** gerontechnology, home support, aging in place, AgeTech, older adults, professional caregivers, family caregivers, seniors

According to the United Nations Department of Economic and Social Affairs (2023), estimates indicate that by 2050, one in six people globally will be aged 65 or older. As the cost of institutional care for older adults continues to rise, it is likely to become increasingly unaffordable for middle- and lower-class families. Also, there is an imbalance between the number of professional caregivers and the number of older adults requiring healthcare. This trajectory seems unsustainable underscoring the need to expand home care options, reserving institutional care for older adults with moderate to severe care needs. Gerontechnology is a relatively young transdisciplinary field established in 1991 to conduct research and development of technological products and services, based on the knowledge of aging processes and to provide innovative tools to contribute to independent living environments, support healthcare and family caregivers, and increase social participation of older adults to promote health, comfort, and safety. Gerontechnology offers promising solutions to support “Aging in place” complementing traditional care models. Many community-dwelling older adults with and without cognitive impairment along with their family caregivers are already using technology to address challenges related to activities of daily living, often without conclusive evidence of its efficacy.

In recent years, a variety of digital and non-digital gerontechnology applications have been developed to support aging in place. These include tools for medication administration (e.g., reminders and alarms), to environmental monitoring (e.g., air quality sensors and cameras), to fall detection systems, and health and activity monitoring (e.g., smartwatches). Most of these promising solutions are either in development or already available in the marketplace. Additionally, the COVID-19 pandemic has further accelerated the development and adoption of technological solutions supporting remote care and communication (e.g., videocalls), and home service delivery (e.g., meals and groceries). This trend has proven to be particularly significant as reducing institutionalization of older adults has become a key objective for governments and caring families around the world. Gerontechnologies, therefore, offer a potential and promising avenue to help older adults maintain autonomy, independence, and social connections while aging in place. Gerontechnology is also expected to ease the burden on family and professional caregivers.

This Research Topic provides a set of papers describing studies that have explored the development, effectiveness, and adoption of gerontechnologies—innovative technologies designed to support older adults—over the past decade. The focus is on research that not only introduces new technologies but also evaluates their real-world application and impact. Specifically, this Research Topic highlights studies that offer evidence-based insights, shedding light on the various stages of technology development and the factors influencing their adoption.

The research spans several key dimensions:

1. **Feasibility:** investigating whether the proposed gerontechnologies can be effectively developed and integrated into everyday life. This includes examining the technological requirements, potential drivers and barriers, and the practicality of implementing these technologies in the homes of older adults.
2. **Usability:** evaluating how user-friendly these technologies are for older adults and their family or professional caregivers. Usability studies often focus on the interface design, the ease of learning to use the technology, and the overall experience of the user.
3. **Acceptability:** assessing the willingness of older adults and their family or professional caregivers to use these technologies. This includes exploring cultural, social, and psychological factors that influence the acceptance of technology in aging populations.
4. **Efficacy:** determining the effectiveness of gerontechnologies in achieving their intended outcomes. For example, studies might measure whether a particular technology improves health outcomes, enhances quality of life, or reduces the burden on family or professional caregivers.
5. **Satisfaction:** gauging the gratification levels of older adults and their family or professional caregivers with the technology. This often involves assessing how well the technology meets user expectations and whether it adds value to their lives.
6. **Early adoption:** exploring the factors that contribute to the early adoption of gerontechnologies by older adults and their family or professional caregivers. Research in this area might examine how early adopters are identified, what motivates them to try new technologies, and the challenges they face in doing so.
7. **Accessibility:** ensuring that gerontechnologies are available to all older adults, regardless of physical, sensory, or cognitive abilities. This involves designing technologies that can be used by individuals with disabilities or impairments or less tech-savvy older adults, and making sure that these technologies are available in different languages, formats, settings, and even forms. Accessibility also includes addressing the economic aspects, ensuring that these technologies are affordable and available to a wide range of older adults.

This Research Topic is particularly interested in studies that employ cross-sectional methodologies, using qualitative, quantitative, or mixed methods approaches. Cross-sectional studies are valuable because they provide a snapshot of the population at a specific point in time, offering insights into the current state of technology adoption and use among older adults and their family or professional caregivers.

The focus is on gerontechnologies that have been tested in real-world settings, particularly within the homes of older adults. This includes studies involving family caregivers, older adults, or both, and covers a broad spectrum of aging experiences—from normal aging processes to more complex conditions like dementia.

Additionally, this Research Topic encompasses various aspects of the technology development continuum:

- **Recruitment:** examining how older adults and family or professional caregivers are recruited for studies on gerontechnologies, which can provide insights into the demographics of early adopters and the generalizability of the findings.
- **Technology development:** focusing on the design and creation of gerontechnologies, including how they are tailored and personalized to meet the needs of older adults and their family or professional caregivers.
- **Early adoption and implementation:** studying the initial rollout and integration of these technologies into everyday life, including the strategies used to encourage adoption and overcome barriers, and learning about the challenges, obstacles, drivers, as well as successes.
- **Public policy:** investigating the role of public policy in supporting or hindering the development and adoption of gerontechnologies. This might include policies related to funding, regulation, and the promotion of technology in aging populations.

Overall, this Research Topic aims to contribute to a deeper understanding of how gerontechnologies can be effectively developed, adopted, and used to improve the lives of older adults and their family or professional caregivers, while also informing future research, development, and policy efforts in this field.

The content can be read following the order suggested here, or be appraised depending on the readers' specific interest in different aspects of gerontechnology. The articles provide empirical data on different challenges and opportunities in gerontechnology research and development.

The first article of this Research Topic addresses recruitment for gerontechnolgy studies and the lessons learned from the pandemic challenges to research. Williams et al. focus on the problem of recruitment of user-caregiver dyads for gerontechnology research in the context of older adults living with dementia. They use COVID-19 as an example of one of the most challenging periods to recruit older adults living with dementia to participate in research using sensors to monitor activities of daily living. With community-based strategies such as the distribution of flyers alongside home-delivered meals, they implemented the least expensive and most effective strategy to recruit their participants. This strategy was possible in collaboration with home-delivered meal programs who are trusted service providers. Also, “Word of mouth” emerged as the second-highest source of dyad enrollment, being a low-cost, relatively low-effort recruitment method. Researchers can benefit from these strategies as older adults are more likely to respond to surveys provided by someone trusted and known to them rather than cold surveys received in the mail or e-mail.

Gerontechnology research is not only challenging in terms of recruitment; the quality of gerontechnology research is also a concern. Moreno et al. provide a 5-year systematic review of the literature of gerontechnologies tested among community-dwelling older adults with unimpaired cognition and their family caregivers to support aging in place. Surprisingly, only 13 gerontechnologies met the study criteria and were classified into four categories: monitoring technologies, communication technologies, daily life assistance technologies, and health information technologies. The results highlighted the benefits and challenges of each gerontechnology. The study also provided recommendations for technology development, implementation, research, and public policy. Considering a global perspective of aging, the study encouraged the early introduction of these technologies before the onset of cognitive decline including personalization based on dyads' needs and easy-to-use co-created solutions respecting their privacy.

The content also includes a remarkable example of multi-country initiatives to develop gerontechnologies for home support and the cultural and technical challenges of international collaborations. Nap et al. conducted a study with an international team to test a co-created Decision Support System connected to multiple assistive technologies for home support among older adults living with dementia, their family and professional caregivers in the Netherlands, Italy, and Taiwan (e.g., sensors, a fall detection device, GPS tracker, medication dispenser, among others). Their technology integrates data of physical activity, eating and sleeping patterns, cognitive functioning, social contacts, and medication intake using a dashboard. Data come from assistive technologies that are selected based on the profile of the individuals with dementia to respond to individual care and support needs. After a 1-to-6-month test period, participants reported perceived added value in their use, which provided a better insight on the status of people living with dementia at home in three different cultural contexts.

The content also features the use of voice assistants that have shown increasing interest and popularity among gerontechnology scientists. Cao et al. studied the factors influencing older adults' acceptance of voice assistants. The qualitative results among older adults suggested that their acceptance depends on both product characteristics (e.g., perceived usefulness, perceived privacy and security risks, perceived enjoyment, perceived benefits for supporting independent living) and personal characteristics (e.g., technological self-efficacy and dispositional resistance to change). Using partial least squares structural equation modeling, they demonstrated that perceived usefulness, perceived enjoyment, and technological self-efficacy positively influenced older adults' behavioral intention to use voice assistants. When older adults see the benefits of voice assistants, when they perceive enjoyment through their interactions, and especially when they trust their capabilities to use them, they will probably accept them.

On the same line of research, pioneer research conducted by Astell and Clayton offer the first study testing gerontechnologies in the oldest old to combat social isolation with voice assistants. In their community case study, the researchers tested the use of smart speakers by older adults of 90 years of age or older living in supported accommodation. Participants reported a sense of presence having a positive impact on their experience of loneliness and social isolation. Interestingly, using voice control and hearing a voice gave them “agency” creating a sense of connection with the device. They discuss their findings in terms of “Digital Prescribing” to tackle well-being outcomes.

Early adoption has been another challenge in gerontechnology research over the years. Profiling early adopters and the characterization of their needs has implications for public policies. Teles et al. address the early adoption of an eHealth platform developed by the World Health Organization to help dyads of individuals living with dementia and their family caregivers in the Portuguese context. This platform provides a freely accessible and self-guided program delivering caregiver education about dementia, caregiving responsibilities, and strategies for coping with common problems in dementia care. Early adopters of this technology were mostly highly educated women in their early fifties taking care of their parents living with dementia. The use of this platform allowed them to obtain a profile based on the psychosocial needs of Portuguese family caregivers of older adults living with dementia, which is essential for planning the organization of healthcare services.

Another challenge in gerontechnology research corresponds to the characteristics of interfaces designed for older adults to facilitate accessibility. Zhou et al. use the example of human-computer interaction interfaces in smart homes to provide insights into the importance of designing technologies adapted to older adults. These products may use interfaces not allowing users to clearly identify the content, with too much information, leading to cognitive load and a negative experience for older adults. Age friendly interfaces for smart homes have to be easy to understand and facilitate the interaction process. Convenience, simplicity, and warmth can reduce unnecessary operations. For instance, the 18 mm function buttons, the up-and-down sliding layout, and the minimalist style received the best subjective evaluation from older adults. As such, personalization, operation guidance, warm reminders, and touch voice are among the characteristics to make them simple, easy to use, and attractive for end users.

Implementation is another major challenge in gerontechnology research. Jutai et al. discussed the process of the implementation of digital health technologies in home care and long-term care for older adults. The results of their scoping review uncovered 10 thematic Research Topics in peer-reviewed research about technologies for assisted living for older adults (e.g., communication, design, economic analysis, ethical considerations, among others). Based on their findings, they recommend a framework to improve the quality of research in this area so that implementation is planned and executed before the design of a technology has been completed.

The last major challenge addressed in this Research Topic of articles corresponds to public policies in gerontechnology research. Genge et al. propose five key messages for policymakers and funders of gerontechnologies inspired by observations from the Canadian AgeTech context. In their opinion, a life course perspective of aging is necessary to address the heterogeneity and evolving needs of older adults. As such, gerontechnologies must respond to older adults' real problems and be integrated as a complement to existing health and social services. In addition, end-user engagement requires the recognition of older adults as experts with lived experiences participating in co-designing gerontechnologies. Consequently, they stress the importance of finding a more flexible approach to developing and testing gerontechnologies. They recommend that policymakers and funding agencies structure their calls to encourage the participation of older adults with diverse life course perspectives, either as project partners, members of an advisory board, or reviewers of funding applications. Also, financial allowance for family caregivers participating in gerontechnology research can reduce barriers to participation (e.g., to cover the cost of respite care). They stress the importance of policymakers and funders promoting Equity, Diversity and Inclusion (EDI) and Gender-Based Analysis Plus (GBA Plus) plans to ensure that research teams and participants are fully representative of the perspectives of diverse individuals throughout the innovation process.

We hope that readers will find the articles in this Research Topic both useful and enlightening. We believe the content will inspire the readers to be aware of the challenges and opportunities in this exciting transdisciplinary field. In conclusion, as institutional care becomes increasingly unsustainable, it is crucial to continuously update our knowledge on evidence-based technologies for home support tested with both older adults and their family caregivers. Healthcare professionals and families are often disadvantaged by lack of clear information on the utility and cost effectiveness of these technologies. Having reliable, evidence-based data on both emerging and existing gerontechnologies is essential for making informed decisions about their recommendation or purchase. Older adults need proof upon which to make an informed decision to invest in a specific technology when needed. Family caregivers need this information to improve their judgment when trying to choose, purchase, and adopt a technology aiming to solve a specific problem at home and have peace of mind when these technologies are used to support their loved ones. Clinicians, health insurance companies, and governments need a pool of evidence-based technological tools that could be recommended to families facing the loss of autonomy and independence of older adults. Researchers and scholars need a base of knowledge for future applied research in gerontechnology. Finally, policymakers and governments need this information to create legislation aiming to protect the public and the end users of these technologies. As a society, we must act quickly and wisely to make sure that the gerontechnologies that we develop and disseminate have a positive and lasting impact on the lives of older adults and their family caregivers. This is a mission that can be accepted by new generations of highly motivated transdisciplinary scientists and trainees inspired by former generations of visionaries. We are all building innovative possibilities with gerontechnologies to help improve the quality of life of our older adults with new opportunities to continue to live fulfilling lives. At the same time, we are creating new possibilities when we face our own aging to contribute to a society where older adults' wellbeing is a priority.
